# Patterns of allergic sensitization and factors associated with emergence of sensitization in the rural tropics early in the life course: findings of an Ecuadorian birth cohort

**DOI:** 10.3389/falgy.2021.687073

**Published:** 2021-08-05

**Authors:** Philip J Cooper, Irina Chis Ster, Martha E Chico, Maritza Vaca, Mauricio L Barreto, David P Strachan

**Affiliations:** 1 Institute of Infection and Immunity, St George’s University of London, London, UK; 2 Escuela de Medicina, Universidad Internacional del Ecuador, Quito, Ecuador; 3 Fundacion Ecuatoriana Para Investigacion en Salud, Quito, Ecuador; 4 Center for Data and Knowledge Integration for Health (CIDACS)-FIOCRUZ, Salvador, Brazil; 5 Population Health Research Institute, St George’s University of London, London, UK

**Keywords:** allergic sensitization, atopy, cohort, risk factors, childhood, tropics

## Abstract

**Introduction:**

There are limited data on emergence of allergic sensitization (or atopy) during childhood in tropical regions.

**Methods:**

We followed a birth cohort of 2404 newborns to 8 years in tropical Ecuador and collected: risk factor data by maternal questionnaires periodically from birth; atopy was measured by skin prick test reactivity (SPT) to aeroallergens in parents, and aeroallergens and food allergens in children at 2, 3, 5, and 8 years; and stool samples for soil-transmitted helminths (STH) from children periodically to 8 years and from parents and household members at the time of recruitment of cohort children. Data on risk factors were measured either at birth or repeatedly (time-varying) from birth to 8 years. Longitudinal repeated-measures analyses were done using generalized estimating equations to estimate an the age-dependent risk of positive SPT (SPT+) to any allergen or mite during early childhood to school age.

**Results:**

SPT+ to any allergen was present in 29.0% of fathers and 24.8% of mothers, and in cohort children increased with age, initially to mite but later to cockroach, reaching 14.8% to any allergen (10.7% mite and 5.3% cockroach) at 8 years. Maternal SPT+, particularly presence of polysensitization (OR 2.04, 95% CI 1.49-2.77) significantly increased the risk of SPT+ during childhood, while household overcrowding at birth decreased the risk (OR 0.84, 95% CI 0.72-0.98). For mite sensitization, maternal polysensitization increased (OR 2.14, 95% CI 1.40-3.27) but rural residence (OR 0.69, 95% CI 0.50-0.94) and birth order (3^rd^ -4^th^ vs. 1^st^ – 2^nd^: OR 0.71, 95% CI 0.52-0.98) decreased the risk. Time-varying exposures to agricultural activities (OR 0.77, 95% CI 0.60-0.98) and STH parasites (OR 0.70, 95% CI 0.64-0.91) during childhood decreased while anthelmintics increased the childhood risk (OR 1.47, 95% CI 1.05-2.05) of mite sensitization.

**Conclusion:**

Our data showed the emergence of allergic sensitization, primarily to mite and cockroach allergens, during childhood in tropical Ecuador. A role for both antenatal and postnatal factors acting as potential determinants of SPT+ emergence was observed.

## Introduction

Allergen sensitization or atopy represents a predisposition of humans to generate IgE responses to biochemically heterogeneous molecules present in the environment. These molecules or allergens are derived from sources including arthropods, pollens, moulds, and foods. Sensitization rates vary between populations according to host genetic factors, the allergens to which a population is exposed, and the presence of environmental exposures that modify the expression of atopy (1). Allergen sensitization can be measured in epidemiological studies either by the presence of IgE-mediated inflammation to allergen extracts *in vivo* by skin prick testing or by detection of specific IgE in blood samples. Atopy is an important risk factor for the development of common inflammatory diseases such as asthma, eczema, rhinitis, and food allergy (1,2).

There are numerous epidemiological studies from both high-income and low and middle-income countries on rates of allergen sensitization in general population samples and in children and adults with and without evidence of allergic diseases (2-4). These studies show that the prevalence and specificities of allergen sensitization varies considerably across populations (5,6). Although studies from tropical settings are less common, most have shown in both population-based and patient-based studies that the primary allergen sensitizers are arthropod species including dust mites and cockroaches that thrive in these warm and humid environments (6). There are few published longitudinal studies of the emergence of allergic sensitization early in the life course from tropical regions (7,8) that have studied the role of antenatal and postnatal host and environmental factors as risk factors for allergic sensitization.

We have shown previously in a birth cohort, from a tropical rural district of coastal Ecuador, followed to 8 years of age, that childhood and or maternal STH parasites protected against the development of mite sensitization at 3 years (9), to perennial allergens at 5 years of age (10), and to any allergen when measured at 8 years of age (11). In the present analysis, we describe the development and patterns of allergic sensitization measured by allergen SPT+ to school age and explore the potential role of a range of relevant ante-natal and post-natal individual and environmental risk factors, with a particular focus on the role of exposures associated with poor hygiene, in determining the emergence early in the life course of SPT+ in this tropical setting.

## Materials And Methods

### Study Area and Population

Detailed methodology of the study objectives, design, follow-up and sample and data collection for the ECUAVIDA birth cohort study are provided elsewhere (12). Briefly, newborns whose families lived in the rural district of Quininde, Esmeraldas Province, were recruited around the time of birth at the Hospital Padre Alberto Buffoni (HPAB) in the town of Quininde between November 2005 and December 2009. The District is largely agricultural where the main economic activities relate to the cultivation of African palm oil and cocoa. The climate is humid tropical with temperatures generally ranging 23-32°C with yearly rainfall of around 2000-3000 mm. Inclusion criteria were being a healthy baby, collection of a maternal stool sample, and planned family residence in the district for at least 3 years.

### Sample and data collection

Children were followed-up from birth to 8 years of age with data and samples including stools collected at baseline during the initial home visit within 2 weeks of birth and at 7 and 13 months, 2, 3, 5 and 8 years of age. Stool samples were collected from mothers during the third trimester of pregnancy and from household members during the initial home visit around the time of birth of the child. Follow-ups were done either by scheduled visits to a dedicated clinic at HPAB or by home visits. At the initial home visit, a questionnaire was administered to the child’s mother by a trained member of the study team to collect data on risk factors, potential confounders, and allergic diseases. Maternal questionnaires were repeated at the time points listed above. Presence of allergic disease symptoms at 8 years of age were defined as described (11,14).

### Stool examinations

Stool samples were examined using four microscopic techniques to detect soil-transmitted helminth eggs and larvae including direct saline wet mounts, formol-ether concentration, modified Kato-Katz, and carbon coproculture (13). All stool samples were examined using all 4 microscopic methods where stool quantity was adequate. A positive sample was defined by the presence of at least one egg or larva from any of the above detection methods. Parasite burdens with *A*. *lumbricoides* and *T*. *trichiura* were quantified as eggs per gramme (epg) of stool using the results of the modified Kato-Katz method and categorized into light, moderate, and heavy intensities using WHO criteria (13) as follows: *A*. *lumbricoides* (light – 1-4,999; moderate – 5,000-49,999; and heavy - >=50,000 epg) and *T*. *trichiura* (light – 1-999; moderate – 1000-9,999; and heavy - >=10,000 epg).

### Allergen skin prick test reactivity

Allergic sensitization was measured by SPTs and done on fathers and mothers of cohort children and on cohort children at 2, 3, 5, and 8 years. SPT on parents used the following allergen extracts: house dust mites (*Dermatophagoides pteronyssinus/Dermatophagoides farinae* mix) (Greer laboratories, Lenoir, North Carolina, USA), American cockroach (*Periplaneta americana*) (Greer), cat (Greer), dog (Greer), grass pollen (9 southern grass mix containing pollen from Bermuda, Kentucky Blue/June, Johnson, Meadow Fescue, Orchard, Perennial Ryegrass, Redtop, Sweet Vernal, and Timothy grasses) (Greer), fungi (New stock fungi mix containing *Acremonium strictum, Alternaria alternata, Aspergillus niger, Aureobasidium pullulans, Bipolaris sorokiniana, Botrytis cinerea, Candida albicans, Chaetomium globosum, Cladosporium sphaerospermum, Epicoccum nigrum, Fusarium moniliforme, Mucor plumbeus, Penicillium chrysogenum (notatum), Phoma betae, Rhizopus stolonifer*, and *Trichophyton mentagrophytes*) (Greer), *Alternaria tenuis* (Greer), *Blomia tropicalis* (Leti Pharma, Barcelona, Spain), and *Chortoglyphyus arcuatus* (Leti) with positive histamine (10 mg/mL) (ALK-Abello, Horsholm, Denmark) and negative saline controls (ALK-Abello). SPT on children used the following 9 allergen extracts (all Greer): house dust mites (*Dermatophagoides pteronyssinus/Dermatophagoides farinae* mix), American cockroach (*Periplaneta americana*), cat, dog, grass pollen (9 southern grass mix), fungi (New stock fungi mix), egg, milk, and peanut, with positive histamine (10 mg/mL) and negative saline controls (ALK-Abello, Horsholm, Denmark). A positive reaction was defined as a mean wheal diameter (mean of longest and orthogonal diameters) at least 3 mm greater than the saline control 15 min after pricking the allergen onto the forearm with lancets (ALK-Abello). Atopy was defined as a positive reaction to any of the allergens tested. Polysensitization was defined as >=2 positive tests to the same panel of 6 allergens which were tested in both children and parents.

#### Anthelmintic treatments

Individuals with positive stools for STH infections were treated with a single dose of 400 mg albendazole if aged 2 years or greater and with pyrantel pamoate (11 mg/kg) if aged less than 2 years, according to Ecuadorian Ministry of Public Health recommendations (14). Pregnant women were offered treatment after the delivery of their child. All treatments were provided free by members of the study team.

### Statistical analysis

The original cohort was designed to study associations between STH infections and allergic outcomes with follow-up to 5 years which was later extended to 8 years (15). SPT+ to any allergen and to mite defined two longitudinal binary outcomes. SPT testing was done at 24, 36, 60 and 96 months of age with some time variation but for purposes of analyses, follow-up dates were considered fixed. We use generalized estimation equations (GEEs) to fit population-averaged models (16,17) for effects of age, child, parental, household, and poor hygiene characteristics. The assumption of the correlation structure was that of unstructured (the most general) (16-18). Adjusted associations of longitudinal outcomes with risk factors were assessed using adjusted odds ratios and their 95% confidence intervals with significance level set to 0.05. ORs derived from these longitudinal models estimated associations between potential explanatory variables and the age-dependent risk of SPT+. Conceptually, the interpretation is equivalent to a cross-sectional OR. Longitudinal ORs allow stratification of the age-dependent risk of infection by categories of predictors - these are non-linear relationships and an OR>1 represents a higher risk of SPT+ associated with that category compared to the baseline category across all ages, assuming no interaction with age. Minimally adjusted models (for age and age^2^) assessed associations of each factor with risk of SPT+. Multivariable models were built using variables with P<0.1 in minimally adjusted models. Among highly correlated variables considered for inclusion in multivariable analyses (Table 1), the variable/s with the smallest associated quasi-likelihood under the independence model criterion (QIC) criterion for GEEs (19,20) on the same data sample were chosen. The QIC criterion is an adaptation of the Akaike’s information criterion (AIC) criterion for GEEs for model choice (18,19). The final most parsimonious model derived on a complete data sample was subsequently fit back to the original data on as many observations as possible.

Longitudinal cohorts are subject to attrition at follow-up. We investigated patterns in missing data for SPT+ to any allergen and carried out sensitivity analyses by generating new outcomes where 1 indicated a missing observation and 0 otherwise. Sensitivity analyses to missing data are available upon request. The GEE estimation is based on missing completely at random assumption (21). However, random effects based on maximum likelihood estimation were also fit and did not produce very different estimates in terms of magnitude or precision of ORs. This type of estimation, more sensitive to distributional assumptions, is made under missing at random assumption (20). Urban-rural residence was defined by geographic boundaries. All statistical analyses were done using Stata 16 (Statacorp, College Station, Tex).

### Ethical considerations

Study protocols were approved by ethics committees in Ecuador (Hospital Pedro Vicente Maldonado, Universidad San Francisco de Quito, and Universidad Internacional del Ecuador) and UK (London School of Hygiene and Tropical Medicine). The study is registered as an observational study (ISRCTN41239086). Informed written consent was obtained from the child’s mother and minor assent was obtained from the child at 8 years.

## Results

### Cohort participants and characteristics

**Figure 1:**
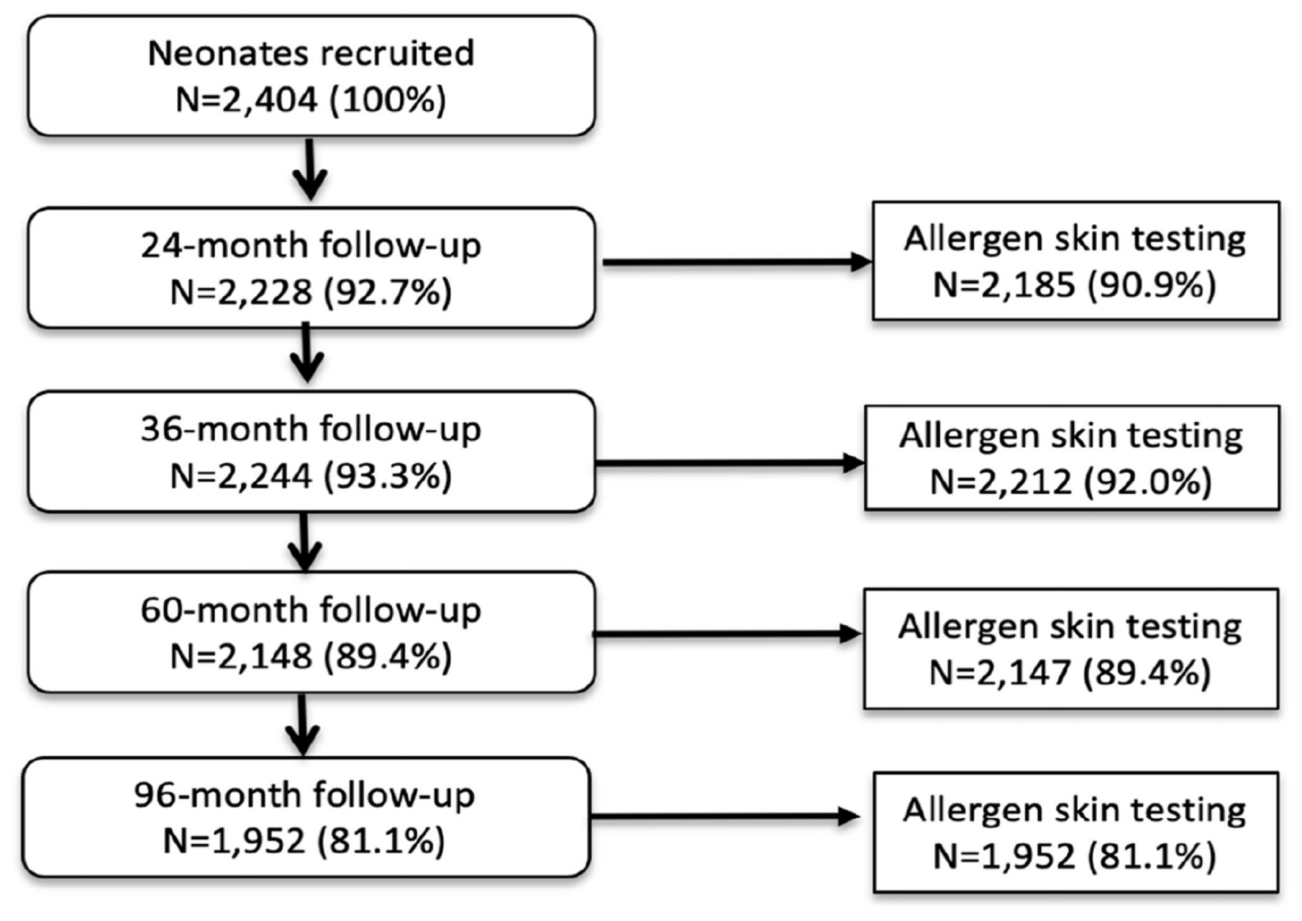
Flow diagram to show follow-up of cohort to 8 years and allergen skin prick testing. Denominator for all proportions is 2,404. A child with any SPT result during follow-up was included in the longitudinal analysis.

Analyses during the first 8 years of life used data from all 2404 children with data for SPT at 1 or more of the 4 observation times from 2 years – 97.0% of children had SPT for at least one observation time and 72.7% had SPT from all 4 observation times. A total of 1,952 (81.2%) had data on SPT at 8 years. Figure 1 shows numbers sampled for SPT between 2 and 8 years. Characteristics of the 2404 newborns at the time of birth (unless otherwise specified) are shown in Table 1. Total numbers of anthelmintic treatments received by the 2,404 newborns recruited over up to 8 years of follow-up were: 0-1 (8.1%), 2-3 (44.1%), and >=4 (47.8%) treatments. Parasite burdens with *A.lumbricoides* and *T*. *trichiura* were predominantly light with a minority having moderate or heavy infection intensities at any specific age between 7 months to 8 years (peak percentage for moderate - 35% for *A*. *lumbricoides* at 30 months and 17% for *T*. *trichiura* at 24 months; heavy - 9% for *A*. *lumbricoides* at 30 months and 2% for *T*. *trichiurai at* 30 months). Proportions with allergic disease symptoms at 8 years among 1,971 children with available data were wheeze (6.6%), eczema (7.9%), and rhinitis (2%).

### Patterns and emergence of allergic sensitization in children and their parents

**Figure 2:**
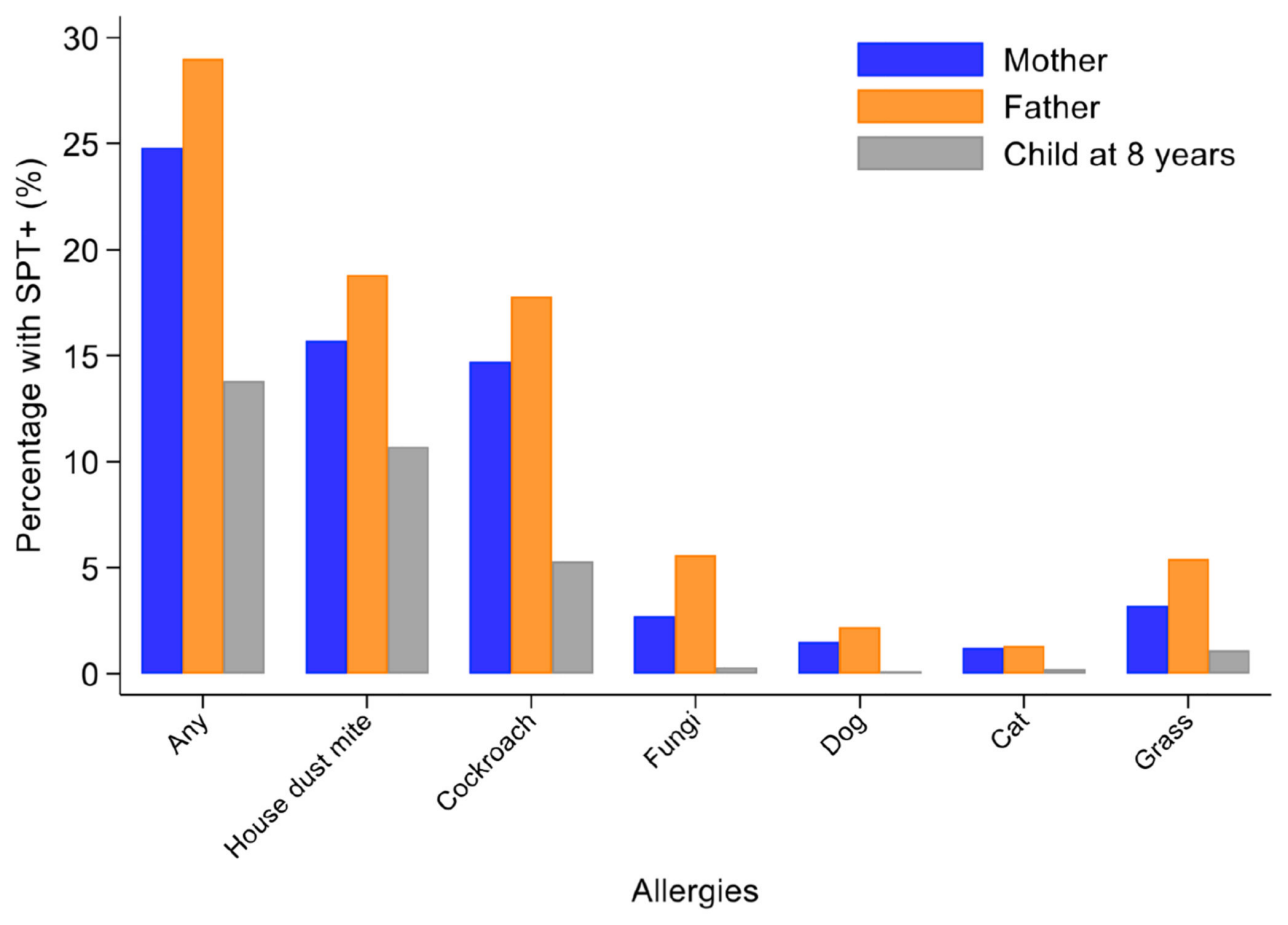
Percentages of mothers, fathers, and cohort children (at 8 years) with positive skin prick tests (SPT+) to any allergen and individual allergen extracts. Data for Mothers (n=2,031) (blue bars), fathers (n=1,191) (orange bars), and children at 8 years (n=1,952) (grey bars).

**Figure 3:**
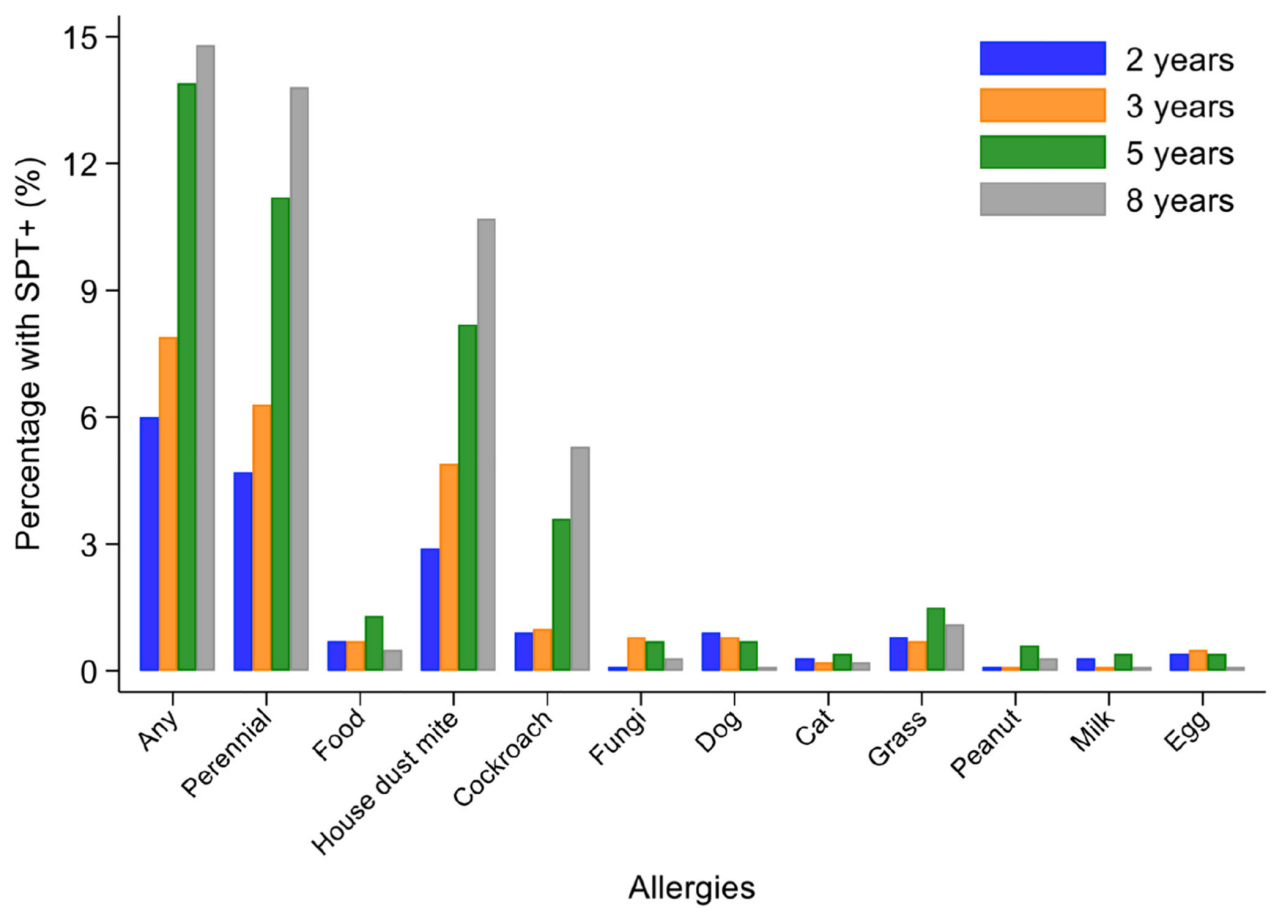
Changes in allergic sensitization profiles between 2 and 8 years of age in cohort. Bars represent 2 (blue), 3 (orange), 5 (grey), and 8 (yellow) years. Perennial allergens included mite, cockroach, dog, and cat allergens.

Percentages of children with SPT+ at 8 years and their parents (2,031 mothers and 1,191 fathers) to the same 6 aeroallergens (*D*. *pteronyssinus/farinae*, American cockroach, dog, cat, fungi, and grass pollens) are shown in Figure 2. Fathers had the greatest rates of sensitization to any of these allergens (29.0%), followed by mothers (24.8%) and cohort children (13.8%). The dominant aeroallergens in this population were *Dermatophagoides* spp. and American cockroach (fathers 18.8% and 17.8%, respectively; mothers 15.7% and 14.7%; and children 10.7% and 5.3%). Allergic sensitization among parents to other aeroallergens were: grass pollens (fathers 5.4% and mothers 3.2%), fungi (5.6% and 2.7%), dogs (2.2% and 1.5%), cats (1.3% and 1.2%), and *A*. *tenuis* (2.6% and 1.5%). Rates of sensitization to allergens not included in Figure 2 were to the mites *B*. *tropicalis* (fathers 6.6% and mothers 4.7%) and *C*. *arcuatus* (6.6% and 3.9%), and to the fungus *A*. *tenuis* (2.6% and 1.5%). Allergic sensitization to the 9 aero and food allergens tested in cohort children from 2 through to 8 years is shown in Figure 3: percentages of children with sensitization to any allergen was greatest at 8 years (14.8%) and was 13.8% to perennial and 0.5% to food allergens. Significant sensitization to individual allergen extracts (>2%) were only seen for *Dermatophagoides* spp. and cockroach: *Dermatophagoides* spp. sensitization was present at 2 years and increased rapidly while cockroach sensitization emerged later at 5 years. Proportions of children with SPT+ to any allergen but without mite sensitization were 50.8% at 2, 37.9% at 3, 41.1% at 5, and 27.4% at 8 years (equivalent proportions for mothers and fathers were 36.7% and 34.9%, respectively). Relatively few children had sensitization to food allergens with rates of <=1.3% (to any of milk, egg, and peanut) at any of the observation times. Polysensitization (sensitization to more than one allergen) increased with age: 2 years (0.7%), 3 years (1.4%), 5 years (2.8%), and 8 years (4.2%). Rates of polysensitization in mothers and fathers were 7.9% and 10.4%, respectively.

### Exposures associated with allergic sensitization to any allergen during first 8 years of life

**Figure 4:**
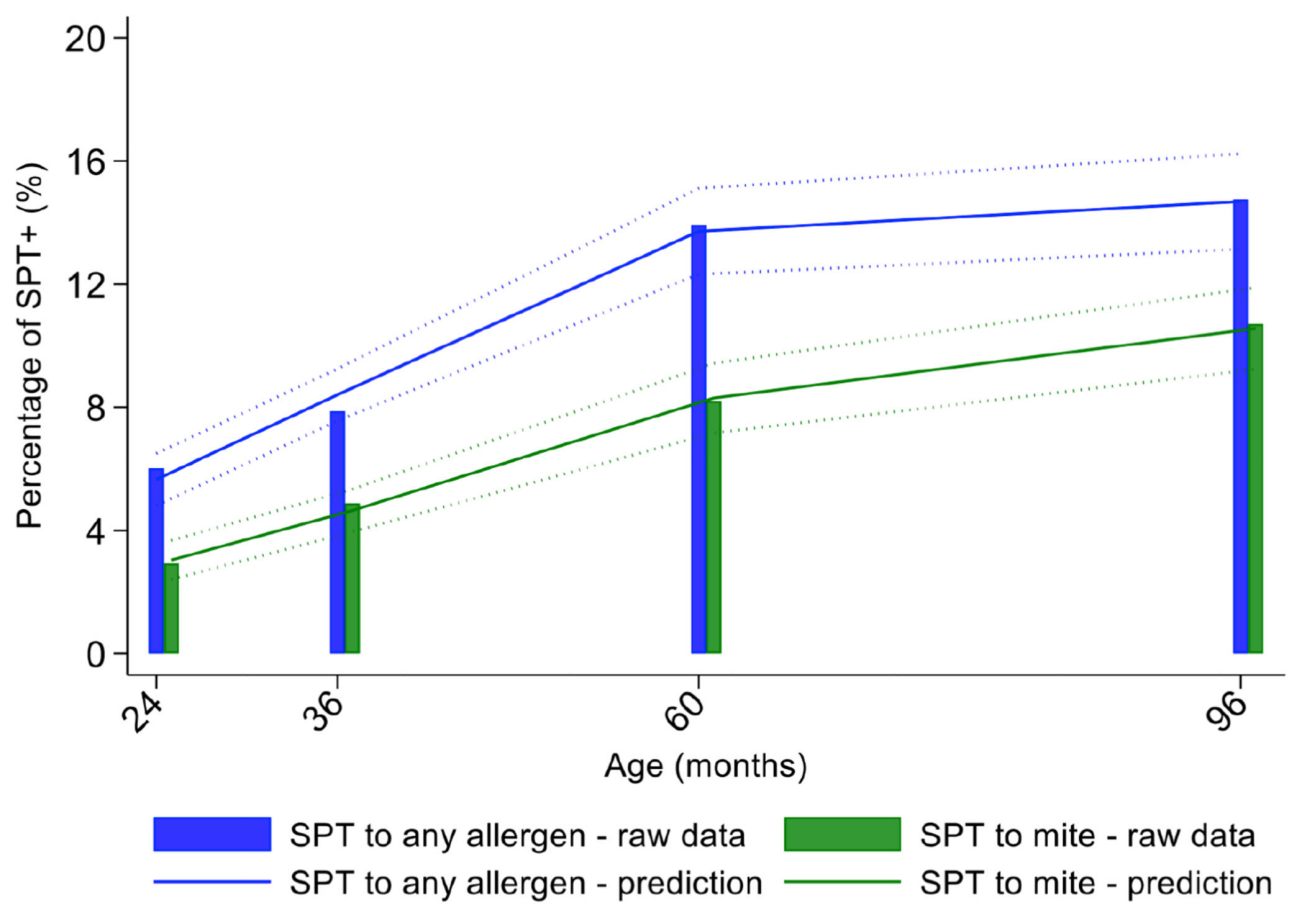
Age-dependent proportions of cohort children with allergen skin prick test positivity (SPT+) to any allergen (blue) and to mite (green). Shown are predictions from population average longitudinal models (predictions shown by solid lines and 95% confidence intervals by dotted lines) against raw data percentages (bars).

**Figure 5:**
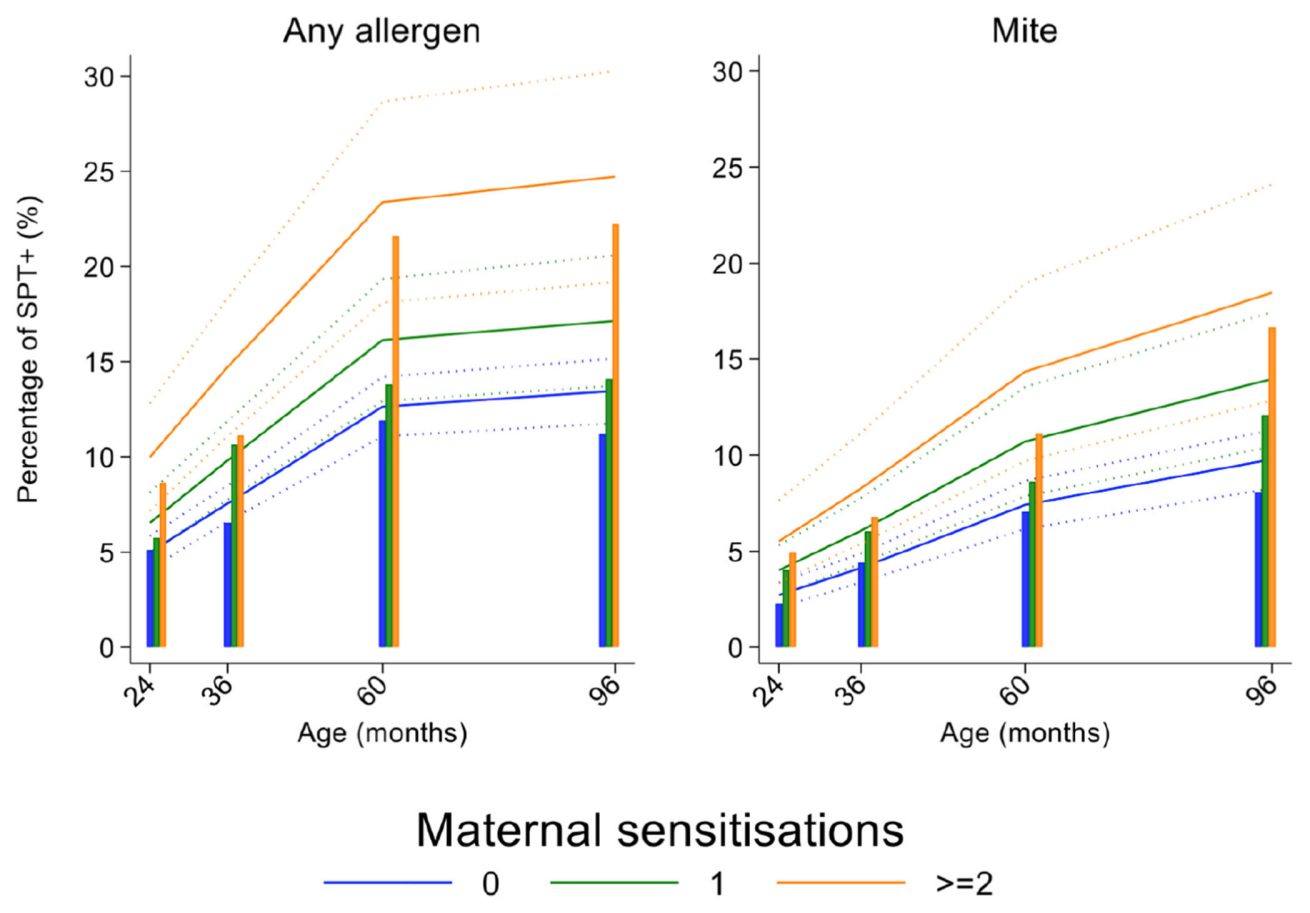
Effects of number of maternal allergen skin prick test positive (SPT+) reactions on age-dependent proportions of cohort children with SPT+ to any allergen and to mite. Shown are predictions from population average longitudinal models (predictions shown by solid lines and 95% confidence intervals by dotted lines) against raw data percentages (bars) stratified by maternal sensitizations. Blue lines/bars – 0 maternal SPT+ reactions; green lines/bars – 1 SPT+ reaction; orange lines/bars – two or more SPT+ reactions.

Longitudinal analyses showed that the risk of SPT+ to any allergen were strongly associated with age in a nonlinear fashion (Table 1 and Figure 4). Minimally age-adjusted population-averaged and multivariable associations between child, parental, household economic and hygiene factors and development of SPT+ during the first 8 years of life are shown in Table 1. Age-adjusted models showed significant positive associations between SPT+ to any allergen and maternal allergic symptoms (OR 1.62, 95% CI 1.12-2.36) and SPT+ (OR 1.57, 95% CI 1.27-1.93) and having a household income greater than $470/month (equivalent to meeting a household’s basic needs) (OR 1.48, 95% CI 1.02-2.16). An increasing number of maternal SPT+ reactions was associated with a greater risk of childhood SPT+ to any allergen (vs. SPT-: monosensitization, OR 1.33, 95% CI 1.03-1.72; polysensitization, OR 2.11, 95% CI 1.55-2.87) Table 1 and Figure 5).

In multivariable analyses (Table 2), childhood SPT+ to any allergen was significantly associated with maternal allergic symptoms (OR 1.54, 95% CI 1.03-2.29) and SPT+ with strong effects seen for more maternal SPT+ reactions (vs. SPT-: monosensitization, OR 1.35, 95% CI 1.04-1.73; polysensitization, OR 2.04, 95% CI 1.49-2.77) (Figure 5) while postnatal household overcrowding was inversely associated with SPT+ (OR 0.84, 95% CI 0.72-0.98).

### Exposures associated with mite sensitization during first 8 years of life

Mite sensitization was strongly associated with age (Table 1 and Figure 4). Age-adjusted analyses showed significant positive associations for mite SPT+ with greater maternal education status (completed secondary vs. illiterate, OR 1.70, 95% CI 1.12-2.59), maternal mite sensitization (OR 1.68, 95% CI 1.28-2.21) and polysensitization (vs. SPT-, OR 2.09, 95% CI 1.40-3.11), having a greater household income (OR 1.72, 95% CI 1.08-2.74), and living in a household constructed with non-traditional materials (OR 1.42, 95% CI 1.04-1.93), while inverse associations were observed for greater period of breastfeeding (7-12 months vs. <=6 months, OR 0.67, 95% CI 0.45-0.99), rural residence (OR 0.70, 95% CI 0.52-0.93), being lower in the birth order (vs. 1^st^-2^nd^: 3^rd^-4^th^, OR 0.75, 95% CI 0.57-1.00; >=5^th^, OR 0.68, 95% CI 0.48-0.96), type of bathroom at time of birth (latrine vs. WC, OR 0.71, 95% CI 0.55-0.91), and STH infections (any household member with STH [OR 0.68, 95% CI 0.48-0.96] maternal STH [OR 0.72, 95% CI 0.56-0.92], and STH infections during childhood [OR 0.70, 95% CI 0.55-0.89]). Interestingly, there was some evidence that anthelmintic treatments given during childhood increased the risk of mite sensitization (OR 1.34, 95% CI 1.00-1.79).

**Figure 6:**
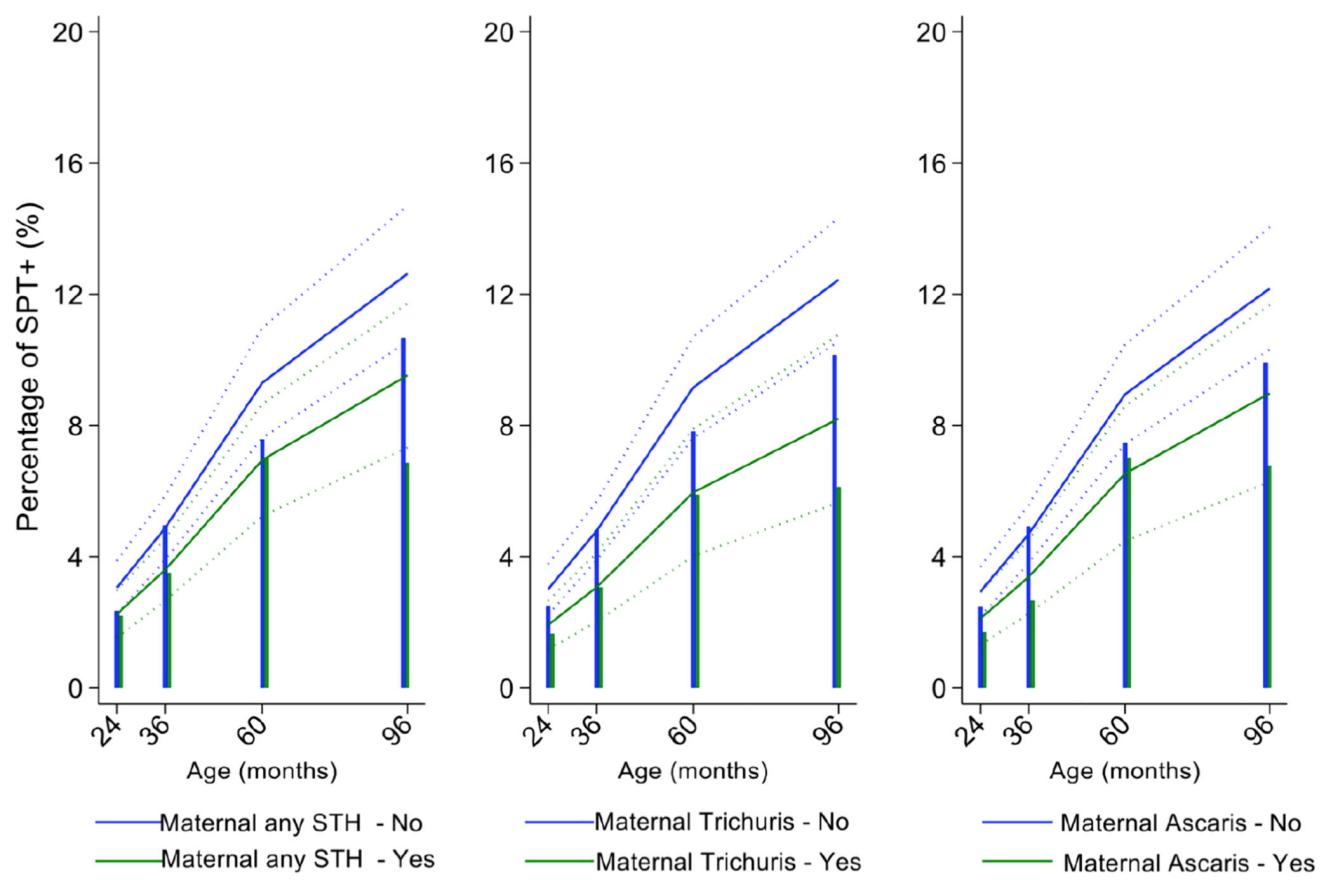
Effect of maternal infections with any soil-transmitted helminth (STH) parasites or with specific STH species on age-dependent proportions of cohort children with allergen skin prick test positivity (SPT+) to mite. Shown are predictions from population average longitudinal models (predictions shown by solid lines and 95% confidence intervals by dotted lines) against raw data percentages (bars). Blue lines/bars – children of uninfected mothers; green lines/bars – children of infected mothers.

**Figure 7:**
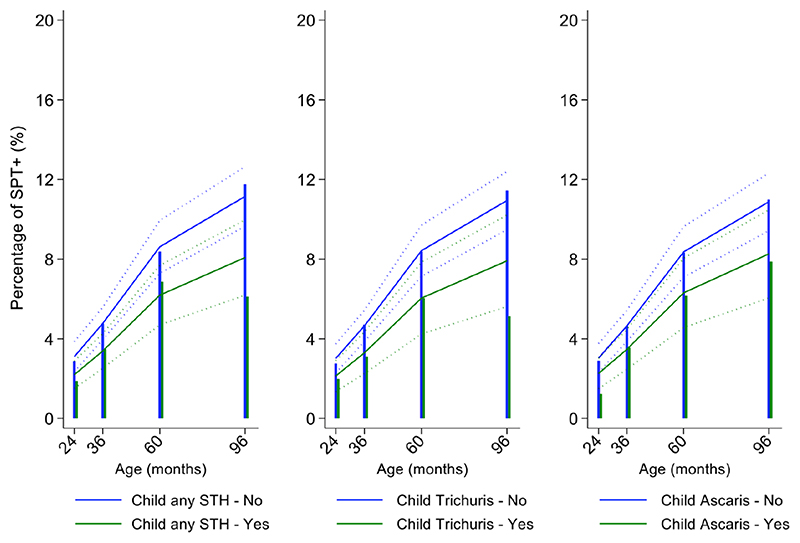
Effect of childhood soil-transmitted helminth (STH) parasites or with specific STH species on age-dependent proportions of cohort children with allergen skin prick test positivity (SPT+) to mite. Shown are predictions from population average longitudinal models (predictions shown by solid lines and 95% confidence intervals by dotted lines) against raw data percentages (bars). Blue lines/bars – uninfected children; green lines/bars –infected children.

In multivariable analyses (Table 2), mite sensitization during childhood remained significantly positively associated with greater number of maternal SPT+ reactions (vs. SPT-: monosensitization OR 1.64, 95% CI 1.17-2.29; polysensitization, OR 2.14, 95% CI 1.40-3.27) (Figure 5) and anthelmintic treatments received during childhood (OR 1.47, 95% CI 1.05-2.05) but inversely associated with rural residence (OR 0.69, 95% CI 0.50-0.94), birth order (3^rd^-4^th^ and >=5^th^ vs. 1^st^-2^nd^, OR 0.71), agricultural exposures (OR 0.77, 95% CI 0.60-0.98), and STH infections acquired during childhood (OR 0.70, 95% CI 0.64-0.91). Maternal STH were not significantly associated with SPT to any allergen or to mite in the adjusted longitudinal analyses. Analysis of effects of parasite type in age-adjusted analyses showed a protective against mite sensitization of maternal and childhood *T*. *trichiura* (maternal – OR 0.63, 95% CI 0.44-0.90, P=0.012; childhood - OR 0.58, 95% CI 0.35-0.98, P=0.042) and childhood *A*. *lumbricoides* (0.74, 95% CI 0.55-0.99, P=0.042) (Figures 6 and 7). There was evidence for stronger protective effects against mite SPT+ of higher parasite burdens with *A*. *lumbricoides* and *T*. *trichiura* during childhood (i.e as a time-varying exposure) although this was statistically significant only for *A*. *lumbricoides* (moderate-heavy vs. uninfected, OR 0.36, 95% CI 0.18-0.72) (Table 3).

## Discussion

In the present analysis, we followed up a birth cohort to 8 years of age in a tropical coastal region of Ecuador to describe patterns and emergence of allergic sensitization in parents and offspring and identify antenatal and postnatal individual and environmental factors that might affect allergic sensitization. Significant proportions of parents had allergic sensitization, mainly to arthropod allergens from mites and cockroaches, while proportions of children with allergic sensitization increased progressively with age, initially to mite but later to cockroach. Rates of sensitization to pollens, pets, fungi, and foods, remained consistently low during childhood. Of the factors we were able to study as potentially associated with allergic sensitization in childhood, maternal atopy was the most important risk factor while protective effects were associated with rural residence, agricultural exposures, and childhood STH infections.

A strength of the study was the prospective design and high rates of adherence to follow-up to 8 years of age of a population-based sample. The longitudinal design allowed us to do a repeated-measures longitudinal analysis for the outcomes (measured at 4 time-points) that provided estimated age-dependent risk of SPT+ in childhood and took into account also time-varying changes in some key environmental exposures such as childhood STH infections. Longitudinal analyses are more informative because they predict the risk of SPT+ at each time point during follow-up, providing an overall age-dependent risk, and insights into the dynamics of SPT+ risk by age during childhood in a manner not possible when outcomes are evaluated at a single point in time. Key outcomes (SPT+) and exposures (STH infections) were measured objectively this minimizing any potential systematic measurement bias. Recall bias is unlikely for risk factor data collected by questionnaire soon after birth for outcomes measured from 2 years of age. We were able collect data on a wide range of potential environmental exposures and potential confounders but cannot rule out potential confounding by unmeasured confounders such as maternal diet during pregnancy. We measured SPT+ to allergens selected as most relevant based on the findings of previous studies done in similar populations in coastal tropical Ecuador (22-24). It is quite possible, by inclusion of a limited panel of allergens, that we underestimated SPT+ in this population. For example, we did not measure SPT+ to *B*. *tropicalis* although a previous study of asthmatic children in the same province showed a much lower rate of SPT+ to *B*. *tropicalis* than to *Dermatophagoides* spp. (25). We were unable to measure SPT+ to pollens largely because of a lack of commercially available and geographically appropriate pollen allergen extracts for the South American region. It has been suggested that clinically relevant pollen sensitization may be less frequent in tropical and subtropical areas than more temperate climates (26). We measured allergen sensitization or atopy using allergen skin prick test positivity rather than measurement of specific IgE. The two measures of atopy are strongly correlated in high-income countries but tend to be dissociated in poorer populations living in low and middle-income countries (27-30), particularly where exposures to parasites such as the soil-transmitted helminth, *A*. *lumbricoides*, are common (6). Helminth parasite infections induce IgE responses to a wide variety of protein and carbohydrate molecules that cross-react extensively with allergens from multiple sources (31-33). Thus, IgE from helminth-infected individuals cross-react extensively with serological assays to detect allergens and allergen components resulting in common false-positive reactions (29,30,34). While it is clear that allergen-specific IgE at high titre from such individuals is strongly associated with allergic symptoms such as asthma (35), the relationship with low titre IgE where most of the IgE-allergen reactivity is seen, is less clear. For this reason, the use of allergen SPT+ is preferable for measuring allergic sensitization in populations living in tropical regions where helminth infections are endemic (30).

We used a birth cohort to define the emergence and development of allergic sensitization during early childhood to school age and measured atopy in parents to estimate rates of atopy in adults. Fathers had the highest rates of atopy (fathers 29% vs. mothers 24.8%) with similar rates of sensitization to mite (*D*. *pteronyssinus* and *D*. *farinae*, 18.8%) and American cockroach (17.8%), and lower rates of sensitization to pollens, fungi, and pets. Patterns were similar in mothers albeit at lower rates. Such patterns of allergic sensitization are consistent with previous data from tropical urban environments (36-38). We saw much lower rates of sensitization (<7%) to the tropical mite, *Blomia tropicalis*, and the storage mite *Chortoglyphyus arcuatus* compared to *Dermatophagoides* spp. *B*. *tropicalis* is an important source of allergens in tropical and subtropical settings and has been associated with respiratory allergies (6,38), atopic dermatitis, and food allergies (39). Mite fauna in house dust samples from tropical settings including Ecuador are highly diverse (6,40, 41) and vary geographically between such settings (6). The storage mite *C*. *arcuatus* has been reported in house dust samples from tropical Latin America (41,42) where sensitization to this mite was observed in patients with asthma (41,43) and allergic rhinitis (41). In this birth cohort, we observed early sensitization to *Dermatophagoides* spp. mites in 2.9% of children by 2 years of age, a rate that increased progressively to 10.7% at 8 years. Cockroach sensitization emerged at 5 years while sensitizations to other aeroallergens were negligible (i.e. <2%). The early emergence of mite sensitization in tropical settings could ‘drive’ international comparisons of atopy, particularly if sensitization to other allergens emerged later in the life course. A bias towards sensitization to invertebrate allergens in early childhood in such a setting is perhaps not surprising given the tendency of children, living in conditions of poverty in precarious circumstances, to spend more time indoors in microenvironments that are increasingly sealed off from much of the animal and plant world and that, in the humidity and heat of the tropics, provide ideal conditions for the proliferation of mites and cockroaches that themselves produce allergens with a proclivity to become airborne. Polysensitization was relatively infrequent in both children (4.2% at 8 years) and parents (<11%). It should be remembered that this was an unselected population-based cohort with no enrichment for children (or parents) with allergic diseases and thus provides data on rates of sensitization in the general population.

A predominance of SPT+ to mite and cockroach allergens has been observed previously in cohorts of young children from tropical regions: 1) a study of 878 children aged 3 years in Ethiopia observed 8.7% positivity to any allergen (5.6% mite and 4.2% cockroach) (44); 2) a study of 132 young Indonesian children showed 19% had positive skin tests to any allergen at 4 years (10% cockroach and 7% mite) while very few (0.8%) had positive tests to food allergens (45); 3) a study of 1170 children followed up to 9 years in Uganda with a subsample of 569 children tested at 3 years showed a positivity at 3 years of 18% to any allergen (*Dermatophagoides* spp. 11%, *Blomia tropicalis* 12%) and at 9 years of 25% to any allergen (*Dermatophagoides* spp. 18%, *B*. *tropicalis* 15%, and cockroach 12% with low positivity rates [<1.5%] to cat, molds, pollens, and food allergens [peanut, egg, and milk] (7); a study of 244 children aged 2-3 years in Colombia showed SPT+ to any allergen to be 22% (12% *B*. *tropicalis*, 4% *D*. *pteronyssinus*, 2% cockroach, 5% cat, and 4% dog) while at 6 years SPT+ was 9% to *B*. *tropicalis*, 8% to *D*. *pteronyssinus*, 5% to cockroach, and <1.2% to cat and dog (8). As for the present study, the latter two studies showed the early emergence of mite sensitization and a a low rate of sensitization to food allergens. There are relatively few studies of food allergy from tropical and sub-tropical regions of low and middle-income countries (LMICs) and there is a perception that food allergy is less frequent in LMICs compared to high-income countries, although current data are considered inadequate for systematic reviews of food allergy risk in LMICs (46,47).

Previous cross-sectional studies done in pre-school and school age children in Ecuador have shown the following: 1) analysis of urban and rural schoolchildren in tropical regions of Esmeraldas Province showed risk of SPT+ to any allergen to be 10.0% (7.6% to mite and 2.2% to cockroach) in urban and 12.5% (6.8% to mite and 4.5% to cockroach) in rural areas (24); 2) analyses of rural school children in sub-tropical and tropical areas of Pichincha Province showed prevalence of SPT+ to any allergen of 20 to 24.0% (mite, 9.3%-8.0%; cockroach 9.4%-15%) in different samples (22,23); 3) analysis of urban and rural schoolchildren in highland Ecuador (altitude >1500 m) showed SPT+ to any allergen of 52.6% (42.1% to mite and 19.4% to cockroach) in urban and 55.9% (43.4% to mite and 28.4% to cockroach) in rural samples (48); 4) analysis of pre-school children aged 3 to 5 years in city of Cuenca in a highland region (>2,000 m altitude) showed SPT+ to any allergen of 33.5% (24.3% to mite and 2.6% to cockroach) (49); and analysis of schoolchildren in Quito in a highland region (altitude 2,800m) aged 6 to 21 years showed SPT+ to any allergen of 34.4% (34.1% to mite) (50). National studies in samples of children have shown, therefore, a predominance of positivity to mite and cockroach allergens using highly standardized extracts from reputable suppliers (ALK, Greer, and Leti) indicating the importance of sensitization to arthropod allergens. Further, SPT+ was much greater in higher altitude areas of the country (i.e. >1,500 metres where climate can vary from sub-tropical to more temperate) than tropical regions of the country despite climatic conditions in tropical areas favouring greater exposures to arthropod-derived allergens. However, significant densities of mites and concentrations of mite allergens have been observed in highland settings such as Quito where humidity is sufficient to support mite growth (41). Such geographic differences in rates of SPT+ are unlikely to be explained by regional differences in ethnicity given a greater level of African genetic admixture in coastal populations that might increase risk (51,52). A more plausible explanation is differences in key allergy-modifying environmental factors. An exposure that is consistently greater in tropical and sub-tropical regions of the country is STH parasite infections that thrive in the warm and moist conditions in tropical areas of the country (53). STH infections have been shown consistently to be inversely associated with SPT+ in different populations in tropical and sub-tropical regions of coastal Ecuador (adjusted ORs varying 0.62 to 0.78 between different studies) (22-24). These observations point to the fact that socio-environmental factors are likely to be more important risk factors for allergic sensitization than allergen-specific factors such as dosage or timing of exposure.

In this analysis, we focussed largely on the role of factors relating to poor hygiene as potential determinants of the expression of allergic sensitization. The only factor that was significantly associated with a reduced risk of SPT+ to any allergen during the first 8 years of life was household overcrowding as observed previously in a cross-sectional analysis in schoolchildren from a neighbouring district (24). Overcrowding could mediate a protective effect against allergic sensitization through increased risk of childhood infections or a more diverse microbiome (54). Environmental factors protective against mite sensitization in the longitudinal analyses included rural residence, birth order, agricultural exposures (defined by living on farm or at least weekly visits to a farm), and childhood infections with STH. Rural residence and farming exposures have been consistently shown to protect against allergic sensitization in a wide variety of settings (55) and may mediate their effects through exposure to more biodiverse environments that provide strong immune regulatory signals (56) during the first 1,000 days of life. The effect of agricultural exposures was dependent on the presence of the exposure during childhood (i.e. time-varying variable) indicating active modulation of SPT+.

Similarly, childhood STH were significantly protective when present early in the life course with stronger effects observed for *T*. *trichiura* than for *A*. *lumbricoides*. These results are consistent with a previous analysis from this cohort of the effects of early life STH exposures on the presence of SPT+ to any allergen at 8 years of age (i.e. an analysis that ignored the effects of age on the acquisition of SPT+) in which protective effects were observed among children of STH-infected mothers. In the previous analysis, strongest protective effects against SPT+ at 8 years were seen among children with STH infections during the first 5 years of life who also had infected mothers (11). Here, using an analysis that estimated age-dependent risk of SPT+ rather than at a single point in time, we observed a significant protective effect of maternal infections with *T*. *trichiura* against mite SPT+ over the first 8 years of life. Further, we observed an effect of STH infections acquired during childhood (i.e. as a time-varying exposure) on mite SPT+ with the strongest protective effects being present in children acquiring *T*. *trichiura* infections who also had infected mothers. These findings could indicate evidence for an antenatal effect of maternal *T*. *trichiura* in reducing age-dependent risk of mite SPT+ but which required infections during childhood to maintain the effect. Potentially, maternal *T*. *trichiura* infections could induce *in utero* modulation of the Th2 immune response of the developing foetus such that the capacity for allergic sensitization in offspring is reduced with infections acquired during post-natal life reinforcing such modulatory effects. We have shown previously that maternal STH can sensitize the foetus to STH antigens (57) and the type of sensitization induced (e.g. for greater immune reactivity or tolerization) is likely determined by the nature of the maternal immune response to that parasite (45,58). Detection of a specific effect on mite SPT+ may relate to the fact that this is the dominant sensitizing allergen in early childhood in this population and to the extensive immunological cross-reactivity between STH and mite allergens (e.g. tropomyosin, paramyosin and glutathione-S-transferase) (6) that could cross-modulate parasite-mite allergen-induced immediate hypersensitivity responses (32).

Anthelmintic treatments given during childhood (i.e a time-varying exposure) increased the risk of mite sensitization supporting a role for childhood STH infections, acquired in early childhood, in the active suppression of mite SPT+. We have shown previously: 1) in a cluster-randomized trial of albendazole given every 2 months to schoolchildren over a period of 12 months that anthelmintic treatment had no effect on SPT+ prevalence (23); and 2) in an observational study of the impact of a mass drug administration programme using the broad-spectrum anthelmintic drug ivermectin to eliminate onchocerciasis, that children living in intervention communities had a greater prevalence of SPT+, an effect was explained best by a lower prevalence of *T*. *trichiura* (59). In the latter study, anthelmintic treatments had been started before the study children were born in intervention communities implying that a reduction in maternal STH infections or transmission in early life (but not at school-age) are critical for mediating the protective effects of STH against SPT+.

We observed a strong association between maternal but not paternal atopy on the development of allergic sensitization during childhood with stronger maternal effects observed with greater number of sensitizing allergens. Previous studies have shown parental history of atopic diseases to be a risk factor for allergic outcomes in offspring (60,61). There are more limited published data on effects of parental allergic sensitization on sensitization in offspring. A study in Germany showed that maternal but not paternal SPT+ was associated with allergic sensitization in children aged 7 to 16 years (62). Maternal SPT+ could increase the risk of atopy in offspring through increased genetic risk, intra-uterine factors, and other maternal factors such as diet. Although heritability of allergen SPT+ has been estimated at 35%, findings of genome-wide association studies for this trait showed no specific associations and indicated that genetic effects were likely mediated through a large number of different genes each contributing only a small risk (63). The effect of maternal but not paternal atopy is less suggestive of genetic or shared environment than specific intra-uterine effects. Children can be sensitized to allergens *in utero* (64) and our data suggest that maternal SPT+, particularly multiple sensitizations (taken as a measure of greater propensity to atopy), may alter how a child’s immune response is programmed to react to allergens during the process of immune development and maturation in early childhood. Such effects could also be mediated through epigenetic mechanisms induced by environmental triggers (e.g. maternal nutrition) occurring during limited time frames *in utero* and early postnatal life (65).

In conclusion, there are limited data from birth cohorts in populations in tropical regions of low and middle-income countries studying the natural history of allergen sensitization and the individual and environmental risk factors for sensitization early in the life course. Here, we followed up a birth cohort to 8 years of age showing that the dominant sensitizing allergens present in this tropical setting are those relating to arthropods, specifically mite (more *D*. *pteronyssinus* than *B*. *tropicalis*) and cockroach, and mite sensitization emerges earliest and predominates to school age. Our data showed a role for both antenatal and postnatal factors in determining the emergence of allergic sensitization in offspring. Maternal SPT+ increased a child’s risk of SPT+ to any allergen indicating the importance of *in utero* factors in determining a child’s long-term risk of atopy. Childhood STH infections and agricultural exposures played an important role in modifying the risk of mite sensitization postnatally even after controlling for maternal atopy. Our data were from a population-based sample and the findings are generalizable to similar populations of children living in rural settings in tropical areas of Latin America. Future prospective studies in tropical LMIC settings could usefully explore the causal link between the acquisition of allergen sensitization and the development of allergic diseases during childhood and how individual and environmental factors modify this link.

## Figures and Tables

**Table 1:** Minimally age-adjusted associations between risk of allergen skin prick test positivity (SPT+) to any and to mite allergens during first 8 years of life and child, parental, household and hygiene factors.

Variable	N (%)	SPT+ to any allergen			SPT+ to mite		
		OR	95%CI	p-value	OR	95%CI	p-value
**Child factors**							
**Gender**							
Male	1193 (51.2%)	1			1		
Female	1139 (48.8%)	0.88	0.73 - 1.06	0.185	0.91	0.72 - 1.17	0.466
**Breastfeeding**							
0-6 months	232 (10.5%)	1			1		
7-12 months	956 (43.3%)	0.93	0.67 - 1.27	0.628	**0.67**	**0.45 - 0.99**	**0.044**
>12 months	1022 (46.2%)	0.91	0.66 - 1.24	0.541	0.74	0.51 - 1.10	0.126
**Maternal factors**							
**Age (years)**							
<=20	622 (26.7%)	1			1		
21-29	1129 (48.4%)	1.14	0.91 - 1.43	0.264	1.23	0.91 - 1.67	0.183
>=30	581 (24.9%)	1.11	0.86 - 1.44	0.425	1.22	0.86 - 1.72	0.268
**Ethnicity**							
Afro-Ecuadorian	599 (25.7%)	1			1		
Non-Afro-Ecuadorian	1733 (74.3%)	0.97	0.79 - 1.20	0.774	0.84	0.64 - 1.10	0.2
**Education**							
Illiterate	353 (15.1%)	1			1		
Primary	1370 (58.8%)	1.17	0.88 - 1.54	0.287	1.31	0.88 - 1.93	0.183
Secondary	609 (26.1%)	1.34	0.99 - 1.82	0.06	**1.70**	**1.12 - 2.59**	**0.013**
**Allergic symptoms**							
No	2204 (95.1%)	1			1		
Yes	113 (4.9%)	**1.62**	**1.12 - 2.36**	**0.011**	1.43	0.86 - 2.37	0.17
**Allergen SPT+**							
No	1528 (75.2%)	1			1		
Yes	503 (24.8%)	**1.57**	**1.27 - 1.93**	**<0.001**	**1.68**	**1.28 - 2.21**	**<0.001**
**Polysensitization**							
0	1528(75.2%)	1			1		
1	345(17.0%)	**1.33**	**1.03 - 1.72**	**0.027**	**1.50**	**1.08 - 2.07**	**0.014**
>=2	158(7.8%)	**2.11**	**1.55 - 2.87**	**<0.001**	**2.09**	**1.40 - 3.11**	**<0.001**
**Paternal factors**							
**Age (years)**							
<=20	188 (8.1%)	1			1		
21-29	1027 (44.0%)	0.82	0.59 - 1.15	0.251	1.06	0.67 - 1.70	0.800
>=30	1117 (47.9%)	0.78	0.56 - 1.08	0.138	0.99	0.62 - 1.57	0.953
**Ethnicity**							
Afro-Ecuadorian	526 (23.3%)	1			1		
Non-Afro-Ecuadorian	1734 (76.7%)	0.89	0.72 - 1.10	0.279	0.81	0.61 - 1.07	0.135
**Education**							
Illiterate	330 (15.5%)	1			1		
Primary	1130 (53.0%)	0.96	0.73 - 1.27	0.795	1.15	0.78 - 1.68	0.486
Secondary	67**2 (31**.5**%)**	1.00	0.75 - 1.35	0.979	1.19	0.76 - 1.73	0.508
**Allergic symptoms**							
No	2074 (96.4%)	1			1		
Yes	77 (3.6%)	0.77	0.44 - 1.35	0.363	0.79	0.38 - 1.66	0.533
**Allergen SPT+**							
No	843 (70.8%)	1			1		
Yes	348 (29.2%)	1.27	0.97 - 1.66	0.083	1.28	0.90 - 1.84	0.173
**Polysensitization**							
0	843(70.8%)	1			1		
1	223(18.7%)	1.33	.98 - 1.82	0.070	1.31	0.86 - 1.99	0.204
>=2	125(10.5%)	1.15	.76 - 1.74	0.495	1.23	0.72 - 2.12	0.444
**Household factors**							
**Residence**							
Urban	1638 (70.2%)	1			1		
Rural	694 (29.8%)	1.00	0.82 - 1.23	0.979	**0.70**	**0.52 - 0.93**	**0.013**
**Monthly income**							
<1 family basket	1950(94.4%)	1			1		
>1 family basket	115(5.6%)	**1.48**	**1.02 - 2.16**	**0.040**	**1.72**	**1.08 - 2.74**	**0.024**
**Material goods**							
1-2	1160 (49.7%)	1			1		
3-4	1172 (50.3%)	1.04	0.86 - 1.25	0.703	1.21	0.95 - 1.54	0.132
**Hygiene factors**							
**Birth order**							
1^st^-2^nd^	1 158 (49.7%)	1			1		
3^rd^-4^th^	725 (31.1%)	0.92	0.75 - 1.13	0.431	**0.75**	**0.57 - 1.00**	**0.049**
>=5^th^	449 (19.2%)	0.85	0.66 - 1.09	0.194	**0.68**	**0.48 - 0.96**	**0.028**
**Daycare to 36 m**							
No	1862 (82.6%)	1			1		
Yes	392 (17.4%)	1.07	0.84 - 1.36	0.593	1.04	0.76 - 1.44	0.804
**Daycare (months)**							
0	1862 (82.6%)	1			1		
1-12	281 (12.5%)	1.20	0.92 - 1.57	0.183	1.18	0.83 - 1.68	0.365
>12	111 (4.9%)	0.76	0.47 - 1.23	0.260	0.71	0.37 - 1.37	0.311
**Crowding (tv)**							
No	NA	1			1		
Yes		0.87	0.75 - 1.02	0.083	0.87	0.72 - 1.04	0.118
**Crowding at birth**							
No	956 (41.0%)	1			1		
Yes	1376 (59.0%)	0.95	0.78 - 1.15	0.594	0.88	0.68 - 1.12	0.292
**House move (tv)**							
No	NA	1			1		
Yes		1.14	0.99 - 1.32	0.071	1.08	0.91 - 1.27	0.390
**House construction (tv)**							
Traditional	NA	1			1		
Non-traditional		1.11	0.91 - 1.36	0.313	1.14	0.89 - 1.47	0.31
**House construction at birth**							
Traditional	598 (25.6%)	1			1		
Non-traditional	1734 (74.4%)	1.10	0.88 - 1.38	0.395	**1.42**	**1.04 - 1.93**	**0.027**
**Potable water at birth**							
No	1527 (65.5%)	1			1		
Yes	805 (34.5%)	1.10	0.91 - 1.33	0.327	1.24	0.97 - 1.60	0.087
**Bathroom (tv)**							
WC	NA	1			1		
Latrine		0.91	0.76 - 1.10	0.342	0.83	0.66 - 1.05	0.118
**Bathroom at birth**							
WC	694 (29.8%)	1			1		
Latrine	1638 (70.2%)	0.85	0.70 - 1.04	0.112	**0.71**	**0.55 - 0.91**	**0.007**
**Dog in house (tv)**							
No	NA	1			1		
Yes		1.14	0.99 - 1.31	0.074	1.06	0.90 - 1.25	0.508
**Dog in house at birth**							
No	1996 (85.6%)	1			1		
Yes	336 (14.4%)	0.97	0.75 - 1.27	0.833	0.97	0.68 - 1.38	0.86
**Cat in house (tv)**							
No	NA	1			1		
Yes		0.99	0.86 - 1.15	0.937	0.98	0.83 - 1.16	0.794
**Cat in house at birth**							
No	1961 (84.1%)	1				1	
Yes	371 (15.9%)	1.02	0.79 - 1.31	0.897	1.06	0.76 - 1.47	0.725
**Farm animals (tv)**							
0	NA	1			1		
1-2		1.00	0.84 - 1.19	0.972	0.93	0.76 - 1.15	0.495
>=3		1.35	0.98 - 1.86	0.066	1.24	0.85 - 1.83	0.271
**Farm animals at birth**							
0	1510 (64.7%)	1			1		
1-2	675 (29.0%)	0.91	0.73 - 1.12	0.353	0.81	0.61 - 1.07	0.134
>=3	147 (6.3%)	0.73	0.48 - 1.12	0.149	0.68	0.38 - 1.20	0.18
**Agriculture (tv)**							
No	NA	1			1		
Yes		0.98	0.83 - 1.16	0.812	0.84	0.68 - 1.03	0.097
**Agriculture at birth**							
No	1121 (48.1%)	1			1		
Yes	1211 (51.9%)	1.04	0.86 - 1.25	0.679	0.95	0.74 - 1.21	0.667
**Insects at 8 yrs**							
0	648(33.2%)	1			1		
1	758(38.8%)	1.04	0.82 - 1.32	0.732	1.00	0.73 - 1.36	0.985
>=2	547(28.0%)	1.25	0.97 - 1.60	0.085	1.12	0.81 - 1.55	0.486
**Ticks in last 12 months**							
No	1488 (76.0%)	1			1		
Yes	470 (24.0%)	.98	0.78 - 1.24	0.871	0.81	0.59 - 1.11	0.181
**STH parasites**							
**Any infected in household**							
No	771 (40.3%)	1			1		
Yes	1144 (59.7%)	0.83	0.67 - 1.02	0.070	**0.68**	**0.52 - 0.89**	**0.005**
**Father**							
No	708 (73.1%)	1			1		
Yes	260 (26.9%)	0.88	0.73 - 1.06	0.164	0.85	0.55 - 1.31	0.454
**Mother**							
No	1249 (53.9%)	1			1		
Yes	1070 (46.1%)	0.90	0.65 - 1.24	0.509	**0.72**	**0.56 - 0.92**	**0.009**
**Any STH in child before 24m**							
No	2089(89.6%)	1			1		
Yes	243(10.4%)	.85	.61 - 1.19	0.340	.92	.60 - 1.41	0.710
**STH child (tv)**							
No	NA	1			1		
Yes		.86	.71 - 1.10	0.110	**0.70**	**0.55 - 0.89**	**0.004**
**Anthelmintics (to 24m)**							
No	1506 (64.6%)	1			1		
Yes	826 (35.4%)	0.92	0.74 - 1.15	0.465	0.94	0.72 - 1.25	0.687
**Anthelmintics (tv)**							
No	NA	1			1		
Yes		1.18	0.93 - 1.49	0.165	**1.34**	**1.00 - 1.79**	**0.051**

Data are for 2,332 children with at least one evaluation for SPT. Odds Ratios (ORs) and 95% confidence intervals (CI) were estimated by fitting age and age^2^-adjusted population-average longitudinal models using generalized estimating equations. Longitudinal binary outcomes were defined by presence/absence of the atopic outcomes in children followed up between 2 and 8 years of age. Models were fit under missing completely at random assumption for unobserved data points. Characteristics are at time of birth of child or time-varying over the course of follow-up. STH – soil-transmitted helminth infections. Anthelmintic treatments – number of maternal reports of at least one anthelmintic treatment during the previous year. Atopy was measured by allergen skin prick test reactivity to a panel of 9 relevant aeroallergens. Parental allergic symptoms – history of symptoms of asthma, rhinitis, and eczema. A household income sufficient to meet the basic needs of 4 persons in 2008 was $480 monthly. Traditional building materials were wood and bamboo. Non-traditional materials were cement, block, and brick. Household overcrowding was defined as 3 or more people per sleeping room. House move represented a change in location of the child’s household since the last follow-up evaluation. Agricultural exposures represented having contact with animals on a farm at least once a week. Haematophagous insects represented presence of types of haematophagous insects (ticks, reduviid bugs, bedbugs, and lice) in the house over the previous year while ticks in the past 12 months represented the child having a documented tick bite during the previous year. Any infected with an STH infection in the household represented any member of the child’s household with a positive stool sample collected around the time of birth of the cohort child. Maternal and paternal atopy were to any allergen or mite allergens, as appropriate. Age effect per month: SPT+ to any allergen – age OR 1.058, 95% CI 1.043-1.072, P<0.001; age2 OR 0.9997, 95% CI 0.9996-0.9998, P<0.001/ SPT+ to mite - age OR 1.057, 95% CI 1.040-1.073, P<0.001; age2 OR 0.9996, 95% CI 0.9995-0.9998, P<0.001. Missing data: maternal allergic symptoms (17) and atopy (301); paternal ethnicity (72), education (200), allergy (81) and atopy (1141); household income (267); daycare (78); haematophagous insects (379) and child exposure to ticks (374); maternal STH (13); paternal STH (1364); household STH (417).

**Table 2:** Multivariable associations between risk of allergen skin prick test positivity (SPT+) to any and to mite allergens during first 8 years of life and child, parental, household and hygiene factors.

Variable	SPT+ to any allergen			SPT+ to mite		
	OR	95%CI	p-value	OR	95%CI	p-value
**Maternal factors**						
**Allergic symptoms**						
No	1					
Yes	1.54	1.03 - 2.29	0.035			
**Polysensitization**						
0	1			1		
1	1.35	1.04 - 1.73	0.022	1.64	1.17 - 2.29	0.004
>=2	2.04	1.49 - 2.77	<0.001	2.14	1.40 - 3.27	<0.001
**Household factors**						
**Residence**						
Urban				1		
Rural				0.69	0.50 - 0.94	0.019
**Hygiene factors**						
**Birth order**						
1^st^ - 2^nd^				1		
3^rd^ – 4^th^				0.71	0.52 - 0.98	0.036
>=5^th^				0.71	0.49 - 1.05	0.085
**Crowding (tv)**						
No	1					
Yes	0.84	0.72 - 0.98	0.032			
**Agriculture (tv)**						
No				1		
Yes				0.77	0.60 - 0.98	0.035
**STH parasite**						
**STH child (tv)**					
No				1		
Yes				0.70	0.64 - 0.91	0.008
**Anthelmintics (tv)**						
No				1		
Yes				1.47	1.05 - 2.05	0.024

Population average multivariable models were fitted to the binary longitudinal outcomes defined by presence/absence of SPT+ in children followed up between 2 and 8 years of age. Estimates were controlled for age and age^2^. Age effects were nonlinear (p<0.001): Models were fit using generalized estimating equations under missing completely at random assumption for unobserved data points. OR – Odds Ratio. 95% CI – 95% confidence intervals. STH – soil-transmitted helminth; tv - time-varying covariates.

**Table 3:** Multivariable associations between risk of allergen skin prick test positivity (SPT+) to any and to mite allergens during first 8 years of life and infection intensities with *A*. *lumbricoides* and *T*. *trichiura*.

STH Parasite	SPT+ to any allergen			SPT+ to mite		
	OR	95% CI	p-value	OR	95% CI	p-value
*A*. *lumbricoides*						
Uninfected	1			**1**		
Light	0.87	0.67-1.14	0.319	0.80	0.58-1.10	0.176
Moderate/heavy	0.78	0.51-1.17	0.229	**0.36**	**0.18-0.72**	**0.004**
*T. trichiura*						
Uninfected	1			**1**		
Light	0.90	0.70-1.16	0.402	0.78	0.57-1.07	0.129
Moderate/heavy	1.07	0.61-1.91	0.807	0.38	0.13-1.11	0.077

Population average multivariable models were fitted to the binary longitudinal outcomes defined by presence/absence of SPT+ in children followed up between 2 and 8 years of age. Estimates were controlled for age and age^2^. Age effects were nonlinear (p<0.001): STH – soil-transmitted helminth; tv - time-varying covariates. Models were fit using generalized estimating equations under missing completely at random assumption for unobserved data points, and adjusted or age, maternal allergic symptoms and polysensitization, area of residence, birth order, crowding (tv), agriculture exposure (tv), and anthelmintic treatment (tv), according to outcome as shown for multivariable models in Table 2. OR – Odds Ratio. 95% CI – 95% confidence intervals. Infection intensity categories were: *A*. *lumbricoides* (light – 1-4,999; moderate – 5,000-49,999; and heavy - >=50,000 epg) and *T*. *trichiura* (light – 1-999; moderate – 1000-9,999; and heavy - >=10,000 epg).

## Abbreviations

AIC: Akaike’s information criterion
CI: confidence interval
GEE: generalized estimation equations
LMIC: low and middle-income country
OR: Odds ratio
SPT: allergen skin prick testing
SPT+: allergen skin prick test positivity
STH: soil-transmitted helminths
Th2: T helper cell type 2
